# Kinship influences sperm whale social organization within, but generally not among, social units

**DOI:** 10.1098/rsos.180914

**Published:** 2018-08-29

**Authors:** Christine M. Konrad, Shane Gero, Timothy Frasier, Hal Whitehead

**Affiliations:** 1 Department of Biology, Dalhousie University, 1355 Oxford Street, Halifax, Nova Scotia, Canada B3H 4J1; 2 Department of Zoophysiology, Institute for Bioscience, Aarhus University, C.F. Møllers Allé 3, Aarhus 8000, Denmark; 3 Department of Biology, Saint Mary's University, 923 Robie Street, Halifax, Nova Scotia, Canada B3H 3C3

**Keywords:** kin selection, social structure, cooperation, matrilineality, relatedness, cetaceans

## Abstract

Sperm whales have a multi-level social structure based upon long-term, cooperative social units. What role kinship plays in structuring this society is poorly understood. We combined extensive association data (518 days, during 2005–2016) and genetic data (18 microsatellites and 346 bp mitochondrial DNA (mtDNA) control region sequences) for 65 individuals from 12 social units from the Eastern Caribbean to examine patterns of kinship and social behaviour. Social units were clearly matrilineally based, evidenced by greater relatedness within social units (mean *r* = 0.14) than between them (mean *r* = 0.00) and uniform mtDNA haplotypes within social units. Additionally, most individuals (82.5%) had a first-degree relative in their social unit, while we found no first-degree relatives between social units. Generally and within social units, individuals associated more with their closer relatives (matrix correlations: 0.18–0.25). However, excepting a highly related pair of social units that merged over the study period, associations between social units were not correlated with kinship (*p* > 0.1). These results are the first to robustly demonstrate kinship's contribution to social unit composition and association preferences, though they also reveal variability in association preferences that is unexplained by kinship. Comparisons with other matrilineal species highlight the range of possible matrilineal societies and how they can vary between and even within species.

## 1. Introduction

Cooperative societies are pervasive in the animal kingdom [1–3]. For these systems to evolve and persist, the benefits to cooperating individuals must outweigh the costs [1,4]. Costly cooperative behaviours between kin are typically explained in terms of kin selection [5,6], which predicts that individuals maximize their ‘inclusive fitness’ by helping relatives. This hypothesis, however, cannot explain cooperation between non-relatives, and often fails to explain observed variation in cooperation between relatives [1]. In such cases, other mechanisms in lieu of kin selection, or in addition to it, are required to explain seemingly altruistic behaviours. Another frequently considered mechanism is reciprocal altruism, in which individuals exchange favours that have a fitness cost [7,8]. However, despite much focused attention on this mechanism, relatively few examples have been firmly demonstrated [9]. Instead, many cases of cooperation may be driven by processes involving by-product benefits, without reciprocation of costly investments [10,11]. For example, if the very existence of more group members is beneficial, in and of itself (e.g. through safety in numbers), and if group members are difficult to gain or replace, individuals can benefit from helping raise and protect the offspring of others in their group—a process referred to as group augmentation [12,13].

To disentangle potential mechanisms driving cooperative behaviours, long-term studies of social relationships and behaviour are required, together with comprehensive genetic sampling for kinship. These types of datasets are rare among mammals, particularly among marine mammals.

The sperm whale (*Physeter macrocephalus*) provides an interesting case study of social structure and cooperation because it has a multi-level cooperative social structure [14]. Female and juvenile sperm whales live in ‘social units’ that are stable over a time frame of years [15,16], from which males disperse before sexual maturity to live primarily solitarily or with other males [17]. Social units sometimes join together to form temporary ‘groups’, which can last hours to days [16]. Social units have only been observed to form groups with other social units that are members of the same ‘clan’ [18,19], with clan being a higher level of social structure composed of social units that share socially learned behaviours, including distinguishable vocal repertoires [20].

The evolution and persistence of cooperative social units and groups in sperm whales have not been explicitly examined, in part owing to the difficulty of conducting the necessary behavioural studies on this long-lived, nomadic and deep-diving species. Calf care, specifically communal defence against predators, is hypothesized as the primary force driving and maintaining social units [17,21], but it is unclear how these cooperative behaviours evolved. Sperm whale social units are often described as matrilineally based [14,22,23], which makes kin selection a logical hypothesis for explaining cooperation. Yet, the degree to which social units are matrilineal is poorly understood.

Owing to the long-term observations required to confidently delineate long-term social units, kinship has typically been studied at the level of temporary groups, which can contain multiple social units [24–26]. Genetic data on social units have been published for only a few social units to date [26–29], with little or no support for matrilineal social units [26,28,29], except in one well-studied social unit in the Caribbean [27]. Most of these assessments considered matrilineality generically and as a dichotomy: that social units are either matrilineal or not [26–28]. In conventional wisdom, ‘matrilineal’ would refer to social units where most females (or all offspring, in species without male dispersion) remain, for life, with their mothers and other close female relatives. However, examining the degree to which social units are composed of maternal relatives (even if not exclusively so) can deepen our understanding of the role of kinship in shaping patterns of cooperation within sperm whale social units.

If kin selection is a driving force for cooperation, we would also expect rates of association among individuals to vary depending on their degree of relatedness. However, in the few cases where social behaviour has been explicitly examined in relation to kinship, results have been mixed, with kinship and social association positively correlated in one study [27], while no clear correlation was found in two other studies [28,30]. These past studies have been limited by small sample sizes (e.g. a single social unit [27,29]), coarse measures of association (e.g. individuals within 8.3 km of each other [28]) or short-term observations (e.g. a single week [29]). Additionally, it is unknown whether association preferences between social units [31] relate to kinship.

Here, through a decadal study of well-known sperm whale social units, we are able to lessen these limitations and address questions regarding the role of kinship in sperm whale social structure with new depth and detail. In this study, we examine patterns of kinship and social behaviour using data for 65 individuals from 12 sperm whale social units from the Eastern Caribbean. We explicitly consider three possible categories along a gradient of matrilineality. We address three primary questions: (i) to what degree are social units matrilineal, (ii) do rates of association between individuals within social units correlate with relatedness, and (iii) does kinship between social units predict association preferences?

## 2. Methods

### 2.1. Field methods

Fieldwork was carried out in an area of approximately 2000 km^2^, off the leeward, western coast of Dominica, in the Caribbean Sea (15.5° N; 61.5° W) from 2005 to 2016 as a part of a longitudinal research project on sperm whale behaviour [15]. Annual field seasons ranged from two to four months in duration, and occurred between January and June, using various research platforms (total effort: 518 days).

Sperm whales were located and followed, visually by observers on deck during daylight hours, as well as acoustically using hydrophones up to 24 h a day [15]. Photographs were taken of the trailing edge of flukes of juveniles and adults [32] and of the dorsal fins of calves [33] for individual identification. In conjunction with these identification photographs, we recorded observations of associations of individuals in clusters [15], with ‘cluster’ being defined as in box 1, as groupings of individuals at the surface in close proximity to each other (less than 40 m) with coordinated behaviour [16].

Box 1. **Key social structure terms.**

*Cluster:* a grouping of individual sperm whales at the surface in close proximity to each other (less than 40 m) with coordinated behaviour [16].

*Social unit:* sperm whales with long-term, stable social relationships, defined as individuals identified within 2 h of each other in at least two different years [15].

*Group:* sets of sperm whales temporarily travelling together for hours or days, which may include more than one social unit. The group's members may aggregate in close clusters while socializing, or spread out across kilometres while foraging [34].

*Clan:* a higher level of sperm whale social structure composed of social units that share socially learned behaviours, including distinguishable vocal repertoires [20].

*Definition of association:* a way to designate whether two individuals are ‘together’, for the purposes of calculating association indices. In this study, we used three different definitions of association: (i) both photo-identified in the same day, (ii) both photo-identified within 2 h of each other, and (iii) in the same ‘cluster’ (as defined above).

*Association index:* a quantification of the proportion of time that two individuals spend ‘together’ (based on definition of association). Two association indices are used in this study: (i) ‘both identified’ [35] and (ii) half-weight index [36].

We used dip nets to opportunistically collect sloughed skin found floating within the flukeprints of individual whales or clusters of whales [37]. In 2015 and 2016, we also collected biopsy skin samples from specific individuals, to fill known gaps in our sample set. We used a 90 lb draw weight crossbow and bolts with 2.5 cm long tips with 0.5 cm circumferences. (See [38] for details.) Skin samples collected from 2005 to 2010 were stored in ethanol (at a concentration of 70% or greater), and samples collected from 2011 onwards were stored in a 20% DMSO solution saturated with salt [39].

### 2.2. Identifications

As in Gero *et al.* [31], identification photographs were assigned quality ratings, and only high-quality photographs were used for assigning final identifications.

In some cases, well-known adults and juveniles could not be photographed when they fluked, because multiple animals fluked synchronously. In such cases, if the flukes of these well-known individuals were confidently observed by S.G., they were recorded as having been identified (407 out of 6938 identifications). Past analyses have demonstrated that patterns of association do not differ when including these identifications [31]. Likewise, well-known calves who were not photographed but were readily identifiable due to distinct dorsal markings that were visible by eye or because they were known to be the only calf in the social unit were also recorded as having been identified (521 out of 2074 identifications).

### 2.3. Measuring association and defining social units

For our analysis, we considered three definitions of association (box 1). First, as our finest spatiotemporal scale of association, individuals in clusters at the surface, and so likely within visual contact and often in physical contact, were considered to be associated. Second, we defined association more loosely as individuals identified within 2 h of each other. Individuals seen within this time frame are likely close enough to be in acoustic contact. Third, we defined association as being identified on the same day, to capture potential avoidances or behavioural coordination occurring on larger spatiotemporal scales. Existence of preferences and avoidances on this scale are reasonable, considering that sperm whales travel an average of 50 km day^−1^ [40] and individuals in a group can spread out across several kilometres while foraging [16].

To quantify the proportion of time that pairs of individuals spent associated, based on the above definitions of association, we used two different association indices: (i) ‘both identified’ [35] and (ii) half-weight index [36]. See the electronic supplementary material for further details on the calculation of these indices.

Social units were delineated as in Gero *et al.* [15], so that they reflect long-term, stable social relationships (see box 1). For our analysis, we also designated social units as genetically ‘well-sampled’ or not. Well-sampled social units were those for which all adult females were included in the genetic analysis (determined based on the availability of genetic samples). These were the social units that we included for analyses examining association preferences within social units relative to kinship.

### 2.4. DNA extraction, quality control and sexing

We extracted DNA from all skin samples using standard phenol–chloroform procedures [41]. After extraction, DNA from all samples was quantified via spectrophotometry, using a NanoDrop 2000 (Thermo Scientific, Waltham, MA, USA), and DNA concentrations were standardized accordingly for use in polymerase chain reactions (PCRs).

To determine the sex of individuals, we amplified a 94 bp fragment of the *ZFX/ZFY* gene [42]. Within this fragment, a *Taq1* restriction site is present in the *ZFX* but not the *ZFY* sequence, due to a fixed difference between the X- and Y-chromosomes. We digested the amplicon, and we size-separated and visualized the post-restriction enzyme PCR product using ethidium bromide and agarose gel electrophoresis to distinguish females (37 and 57 bp fragments only) from males (37, 57 and 94 bp fragments) [42].

Sperm whale sloughed skin samples vary greatly in the amount and quality of DNA they yield [42], and DNA quantification via spectrophotometry can overestimate the amount of viable DNA in these samples, because it includes fragments that are too short to be amplified in PCRs. Therefore, we also used the results of this sexing reaction as a first stage of quality control, to screen for samples that were degraded beyond being useful to this study. Samples that failed to amplify at the 94 bp *ZFX*/*ZFY* gene fragment were deemed too degraded for subsequent attempts at genotyping or sequencing. Additionally, we used this sexing assay to determine and optimize DNA amplifiability for downstream genotyping [42]. We adjusted DNA concentrations of sloughed skin samples, in proportion to the brightness of the sample's amplified *ZFX*/*ZFY* gene fragments relative to those of a biopsy sample, to maximize success of amplification across microsatellite loci [42]. For example, a sample with *ZFX/ZFY* gene fragments half as bright as those of a biopsy sample would be assigned a functional concentration that is half of the concentration which was determined via spectrophotometry, and thus we would use twice as much template DNA in the subsequent amplification reaction for that sample. Samples that still genotyped poorly (genotyped at less than 10 microsatellite loci; see below) were excluded from further analysis.

### 2.5. Microsatellite genotyping

We amplified DNA samples at 18 microsatellite loci. For PCR conditions and genotyping methods, see the electronic supplementary material. Other loci were also screened for amplification success with sperm whale skin samples but did not produce usable results and were excluded from our analysis (see the electronic supplementary material, table S1).

To address issues associated with low-quality DNA, particularly allelic dropout [43], we applied a multiple-tubes PCR approach. This allowed us to determine rates of genotyping errors and improve confidence in genotypes. For 17 samples, selected at random with respect to DNA quality and quantity, we performed at least two independent PCRs for apparent heterozygotes and seven independent PCRs for apparent homozygotes. These numbers of replicate PCRs were selected based on the conservative approach described by Taberlet *et al*. [44]. This process of multiple amplifications was repeated in its entirety for seven of the samples, with the identities of these samples masked, so that they were blind controls. We determined genotyping error rates by comparing the genotypes of the blind controls to their counterparts and calculating the rate of discrepancies. Using these rates, we determined the number of tubes required to reach a minimum desired level of confidence in genotypes of 99% per locus, and we performed this number of reactions to achieve this level of confidence. If scores from replicate reactions for an individual were inconsistent, additional reactions were performed until one genotype score emerged as at least 100 times more likely (based on above error rates) than the other observed scores. If this likelihood ratio was not achieved in a reasonable number of reactions, no data were included in the analysis for that individual at that locus.

Previous work on sperm whales has demonstrated the absence of significant population differentiation at microsatellite loci within the North Atlantic [45]. Therefore, all genetic individuals sampled off Dominica were considered to be from a single population for the purposes of calculating allele frequencies.

We tested for linkage disequilibrium using GENEPOP v. 4.2 [46], and tested for null alleles and deviation from the Hardy–Weinberg equilibrium using Cervus 3.0.7 [47].

### 2.6. mtDNA haplotype sequencing

To determine mitochondrial DNA (mtDNA) haplotypes, we amplified and sequenced 346 bp at the 5′ end of the mtDNA control region, using the primers t-Pro and Primer 2 [48]. Mitogenomic diversity is relatively low in sperm whales, compared to estimates for other mammalian species, but out of partitions of the sperm whale mitogenome that have been compared, nucleotide diversity was greatest in the control region [49]. For amplification and sequencing reaction conditions and methods, see the electronic supplementary material.

### 2.7. Identification of genetic individuals

To assign whether or not samples with the same or very similar microsatellite genotypes were from the same individual, we estimated the probability of these pairs of samples originating from the same individual (*P*_Ind_), while incorporating genotyping errors (as determined above), and the probability of the two samples being from full-siblings (*P*_Sib_; *sensu* [50,51]). We classified samples as from the same individual if log_10_(*P*_Ind_/*P*_Sib_) was greater than 3, and we classified them as from different individuals if log_10_(*P*_Sib_/*P*_Ind_) was greater than 3. For pairs of samples where neither criterion was met, the sample with the less complete genotype was excluded from further analysis. We also checked the conclusions of this analysis for consistency with mtDNA haplotypes, sex and photographic field identifications. We also calculated a probability of identity for unrelated individuals and for full-siblings for the entire dataset, using a custom R script.

Genetic identities were linked to photo-identities directly when a biopsy sample was collected from a photo-identified whale or a sloughed skin sample was collected from a photo-identified whale, with no other whales in the immediate vicinity. When sloughed skin samples were collected from clusters containing multiple individuals, the sample was assumed to be from any of the whales in the cluster. If all individuals in the cluster except one could be excluded as providers of the skin (based on sex or mismatching microsatellite genotypes with other known samples) then the sample was deduced to be from the remaining individual. If multiple samples collected from different clusters were matched as the same genetic individual, the photo-identities of the whales that were present in all of these clusters were used to aid deduction. For some genetic individuals, more than one photo-identified individual remained non-excluded. These genetic individuals were not used in individual-level analyses, but if all non-excluded photo-identified individuals were from the same social unit, the genetic individual was assigned to this social unit and used in social unit-level analyses. Individuals were also excluded from further analyses if they were not members of known social units or if the photo-identity of the genetic individual could not be deduced, such as when clusters contained unidentified individuals.

### 2.8. Age class

Age classification of social unit members was accomplished based on observations of size and nursing in the field, as in Gero *et al.* [15], combined with inference based on sex assignment. Individuals were classified as either adult females, juveniles or dependent calves. The category ‘juveniles’ included individuals that were noticeably smaller than adult females, but no longer nursing (see [52] for a description of nursing behaviour). Additionally, because mature males are notably larger than adult females [17,53], individuals that were indistinguishable from adult females based on size but sexed as male were also classified as juveniles. Dependent calves were small individuals that were observed nursing. Some individuals that were initially classified as dependent calves were re-classified as juveniles in subsequent years if they were no longer observed nursing.

### 2.9. Assigning maternity and determining likely genetic relationships

To infer maternity of juveniles and dependent calves, we used a full-maximum likelihood method for polygamous diploids implemented in Colony 2.0.6.2 [54]. We based error rates on the final genotyping error rates estimated for our multiple-tubes PCR approach (0.16% for allelic dropout rate and 0.1% for other errors). We performed a set of three runs, to increase the chances of finding the configuration of relationships with the maximum likelihood, and repeated these runs with two different random seed numbers, to confirm the repeatability of the results. All adult females were included as putative mothers, and individuals classified as juveniles or dependent calves were included as offspring. No putative fathers were included. One juvenile female observed throughout the 12-year study period was assumed to be mature by the end of the study period (based on pregnancy ages reported in [53]). Therefore, the runs were performed in replicate with this individual as a putative mother instead of an offspring, but maternity assignment results did not change. We assigned maternity if the female had a mean probability greater than 90% across all runs. Maternity assignments were checked for agreement with mtDNA haplotypes. Individuals were classified as maternal half-siblings if they were assigned the same mother.

To test hypotheses about relationships between adult females, where relative age is unknown, we used the program ML-Relate [55]. We evaluated which relationships (out of parent–offspring, half-sibling/grandmother–granddaughter, full-sibling and unrelated) were consistent with the genetic data at the 0.05 level of significance, by calculating likelihood ratios and using simulations to reject unlikely relationships. If multiple relationships were consistent with the genetic data, this method was also used to identify the most likely relationship.

### 2.10. Determining pairwise relatedness

To estimate relatedness between individuals, we used the R package *related* [56]. Performance of different relatedness estimators varies depending on the relatedness structure of the population, and no single estimator performs best across all relatedness structures [57,58]. Therefore, to select the best estimator for our dataset, we used a comparative function in *related* that uses our population allele frequencies to generate virtual pairs of individuals with specified genetic relationships, and to estimate the relatedness of these pairs using four different relatedness estimators [59–62]. For use in subsequent analysis, we selected the estimator with the highest correlation between observed and expected relatedness values, which was Wang's estimator [62]. We used this estimator to calculate pairwise relatedness values for all pairs of individuals.

### 2.11. Testing relationships between haplotype sharing, pairwise relatedness and association

Across all identified individuals from known social units, we tested for matrix correlations between measures of genetic similarity and social association. A large proportion of pairs of individuals were never both identified in the same time period, leading to many cells with no data in the matrices of social association, which rendered Mantel tests [63] inappropriate for obtaining reliable *p*-values. Instead, we calculated standard analytical *p*-values based on matrix correlation values (excluding dyads with missing data in the association matrix), which, while not strictly valid for matrix data (the assumption of independent observations is not met), provide an approximate indication of statistical significance. The measures of genetic similarity used were mtDNA haplotype sharing (0 or 1) and pairwise relatedness. The measures of association used were: (i) same cluster, in 6 h sampling period, (ii) same cluster, in a year sampling period, (iii) identified within 2 h, in a 10-day sampling period, and (iv) same day, in a year sampling period. To remove the effect of mothers associating with their dependent calves, we omitted the pairwise data for mothers associating with their calves from all analyses. We repeated the analyses with only data for pairs of individuals in the same social unit included.

Similarly, across all genetic individuals that were assigned to a known social unit, we tested for a matrix correlation between pairwise relatedness and shared social unit membership (0 or 1), by performing Mantel tests [63], using SOCPROG2.7 [64]. We also examined the distributions of pairwise relatedness values within and between social units.

### 2.12. Composition of well-sampled social units

For well-sampled social units, we determined the proportions of relationships classified as mother–offspring, second-degree relatives (half-sibling or grandparent–grandoffspring) or more distantly related. We classified individuals as a mother–offspring pair if they were assigned as such based on maternity assignment in colony or if parent–offspring was the most probable relationship in ML-Relate. We classified individuals as second-degree relatives if they could be inferred as such based on mother–offspring relationships or if second-degree was the most probable relationship in ML-Relate. All other pairs were classified as more distantly related, which could also include unrelated individuals.

### 2.13. Categories of matrilineality

To assess the degree to which social units are matrilineal, we defined three possible categories of ‘matrilineal’: strictly matrilineal, generally matrilineal and matrilineally based. Most stringently, a social unit could be categorized as ‘strictly matrilineal’ if all members have a common maternal ancestor who is still living in the social unit. By this definition, social units are expected to split after the death of their common maternal ancestor, but could contain several generations (female sperm whales can live into their 80s [16] and may first conceive at around 9 years of age [53]). At a coarser scale, a social unit could be categorized as ‘generally matrilineal’ if members have a relatively recent common maternal ancestor, who need not be alive. In such cases, social units should have a common mtDNA haplotype and an average genetic relatedness that is above that of the population. A social unit that is not strictly or generally matrilineal could still be considered ‘matrilineally based’ if it is made up of two or more strictly or generally matrilineal families. We assessed which of these categories were consistent with the social and genetic data for the social units in this study.

### 2.14. Within-social unit association

Within each well-sampled social unit, we performed Mantel tests [63], using SOCPROG2.7 [64] to test for significant matrix correlations between pairwise relatedness and association in clusters, at two sampling periods—2 h and a day.

Within the social unit with the most sampled members (unit A), we also examined social modularity in relation to within-social unit genetic structure. We defined social modules such that association indices (based on association in clusters in a daily sampling period) were generally high among individuals in the same module and generally low among individuals in different modules. We used an eigenvector-based method, as suggested by Newman [65], and implemented in SOCPROG2.7 [64] to test for the presence of meaningful modularity (modularity values greater than approximately 0.3) and to delineate the modules. To account for demographic changes, we examined modularity within three different years that span the study period (2005, 2010 and 2015). We examined the congruence between the social modules identified by this method and the matrilineal clusters defined by mother–offspring relationships.

### 2.15. Between-social unit association

For social units for which at least three members were included in the genetic analysis, we tested for relationships between social association and genetic similarity. If one or more members of each of two social units were associated in a sampling period, then those individuals' social units were considered associated in that sampling period. We used four measures of association: (i) same cluster, in 2 h, (ii) same cluster, in a year, (iii) identified within 2 h, in a day, and (iv) same day, in a year. For measures of genetic similarity, we classified each pair of social units' mtDNA haplotypes as same or different, and calculated mean relatedness values. As a measure of recent common maternal ancestry, we calculated mean relatedness values between social units, by averaging the pairwise relatedness values between all pairs of individuals across each pairwise combination of social units.

We performed Mantel tests [63], using SOCPROG2.7 [64] to test for matrix correlations between each index of association and each measure of genetic similarity. One pair of social units appeared to be contributing strongly to correlations, and so the tests were repeated with pairwise data for that dyad omitted.

## 3. Results

### 3.1. Association dataset

On average, individuals were identified in 54 different 2 h periods (range: 5–192), 26 days (range: 3–90) and 4.7 years (range: 1–10). On average, social units were identified in 131 different 2 h periods (range: 31–401), 45 days (range: 12–117) and 6.8 years (range: 2–10). For additional details, see the electronic supplementary material.

### 3.2. Microsatellite dataset and quality control

Out of 153 samples (94.8% sloughed skin and 5.2% biopsy samples), 30 were excluded by quality control (one biopsy and 22 sloughed skin samples failed to sex, seven sloughed skin samples failed to genotype at a minimum of 10 microsatellite loci). After consolidating duplicates (as determined by log_10_(*P*_Ind_/*P*_Sib_) values) and excluding three likely duplicate samples that did not meet the log-likelihood ratio criteria, 95 unique individuals remained, 88.4% of which were scored at all 18 microsatellites, and all of which were scored at no fewer than 16 microsatellites. The mean allelic diversity was 9.3 (range: 3–17) and the mean observed heterozygosity was 0.75 (range: 0.52–0.93). See the electronic supplementary material, table S2 for locus-specific allelic diversity and heterozygosity.

We calculated the total per-allele genotyping error rate for apparent heterozygotes (*E*_het_) to be 1.1%, incorporating contamination and spurious alleles (1.0% collectively) and manual scoring errors (less than 0.1%). For apparent homozygotes (*E*_hom_), the mean error rate was 2.9%, incorporating allele dropout (2.82%) and manual scoring errors (less than 0.1%), but dropout rate varied widely across samples (max = 11.6%). These rates are comparable to those reported previously for sperm whale skin samples (e.g. per-allele microsatellite error rate of 1.27% [66]). Based on our error rates, for apparent heterozygotes, a minimum desired level of confidence in genotypes of 99% per locus was reached with two tubes (compound error rate = 0.013%). For apparent homozygotes, this level was reached with two tubes based on the average dropout rate (compound error rate = 0.085%), but three tubes were required based on the sample with the highest dropout rate (compound error rate = 0.16%). Thus, we performed a second reaction for loci at which an individual appeared heterozygous, and, to account for low-quality samples, we performed at least three reactions for loci at which an individual appeared homozygous.

No loci showed strong indications of null alleles (all frequencies less than 0.05), and we detected no evidence of deviations from the Hardy–Weinberg equilibrium; however, it is challenging to reliably detect null alleles [67]. Two pairs of loci had evidence of linkage disequilibrium after a Bonferroni correction, but given that our dataset is composed of social units of related individuals, this was not unexpected, and it would be difficult to distinguish true linkage from effects of the similarity of genotypes of relatives. Therefore, we did not exclude any loci from the analysis. Probabilities of identity for this dataset were 2.7 × 10^−19^ for unrelated individuals and 1.5 × 10^−7^ for full-siblings.

After exclusion of unidentified individuals and individuals that were not members of known social units, 65 genetic individuals remained that were assigned to 12 known social units and were used in the social unit-level analyses (table 1). Of these, 55 were linked to single photographically identified individuals from those social units and were used in the individual-level analyses (table 1). Six social units qualified as well sampled, with genetic data for all adult females (and at least 70% of all unit members, when calves and juveniles were included), and these social units were included in the within-social unit analyses (table 1).

**Table 1.** Composition and mitochondrial haplotype (mtDNA Hap) of 12 social units sampled off Dominica. Social units were delineated as in Gero *et al.* [15]. Composition includes past and present sampled members. Well-sampled social units (those for which all adult females were genetically sampled) were used for intra-social unit analyses, and are listed in the top section of the table. The number of genetically sampled social unit members includes only those linked to a single identified individual. The number listed in parentheses counts all sampled social unit members, including samples for which individual identity was unknown. For well-sampled social units, the mean relatedness (mean *r*) was calculated according to Wang [62]. Members of these social units were also categorized as adult females (A_F_) or offspring (O). Mother–offspring (1°) relationships were determined using Colony and ML-Relate. Second-degree (2°) relationships were determined using ML-Relate, or inferred-based shared 1° relatives.

social unit	social unit members		sex		mtDNA Hap	mean *r*	age class		relationships (%)		
	known	sampled	F	M			A_F_	O	1°	2°	greater than 2°
A	12	12	9	3	BB	0.137	4	8	15.2	12.1	72.7
F	10	9	5	4	A	0.232	5	4	16.7	25.0	58.3
J	6	5	5		A	0.136	3	2	20.0	0	80.0
R	10	7	6	1	A	0.106	5	2	14.3	14.3	71.4
S	4	3	3		A	0.212	3		33.3	0	66.7
U	4	4	3	1	A	0.333	2	2	33.3	33.3	33.3
C	6	1	1		A	total	22	18	16.9	15.5	67.6
D	7	(4) 2	3	1	A						
N	9	(8) 5	7	1	A^a^						
P	9	(3) 1	1	2	BB						
T	9	(6) 4	6		A						
V	12	(3) 2		3	A						
total	98	(65) 55	49	16	49 A, 15 BB						

^a^Haplotypes for this social unit were obtained for seven of eight samples.

### 3.3. Mitochondrial haplotypes

For mtDNA haplotype assignment, no errors were detected in blind replicates (*n* = 14) nor any inconsistencies for pairs of samples determined to be from the same individuals based on multi-locus microsatellite genotypes (*n* = 7). Haplotypes were successfully sequenced for 61 of 65 sampled social unit members. For samples from three calves, which failed to sequence successfully, haplotypes were inferred based on the haplotypes of their mothers (because we found consistent agreement in the mtDNA haplotypes of all other calves and their genetically assigned mothers, see below).

Two mtDNA haplotypes (A and BB) were identified in individuals from known social units, both of which have been previously observed in the western North Atlantic Ocean [66]. These haplotypes differ by a single nucleotide substitution. This low level of mitochondrial diversity is consistent with previous observations on a global and mitogenome-wide scale [66].

Mitochondrial haplotypes were consistent within social units (which is a requisite result for the social units to be considered matrilineal), though each haplotype was shared by multiple social units. Haplotype A was much more common, being shared by 10 out of 12 social units (table 1).

### 3.4. First- and second-degree relationships

We classified 30 individuals as adult females and the remaining 25 individuals as offspring. Thirteen of these females were assigned as the mothers of 18 offspring; in all cases, the assigned mother was from the same social unit as the offspring. Ten females were assigned to a single offspring each, two were assigned to two offspring each and one to four offspring. These maternity assignments were in agreement with the mtDNA haplotypes of mothers and their offspring, when both had been successfully sequenced. Seven offspring did not have mothers identified from the sampled females. Average pairwise relatedness between identified mother–offspring pairs was 0.52 (range: 0.42–0.67, *n* = 18) and for half-siblings inferred based on shared maternity, average pairwise relatedness was 0.32 (range: 0.12–0.50, *n* = 8).

**Figure 1.**
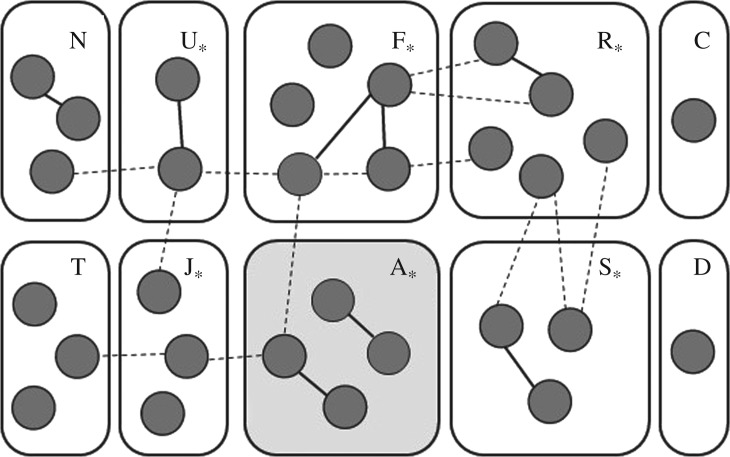
Genetic relationships between adult females, within and between social units. Letters indicate social unit. Shading of social unit block indicates mitochondrial haplotype (unshaded: haplotype A; grey shading: haplotype BB). Solid edges between individuals denote mother–offspring relationships, and dashed edges indicate second-degree relationships, as determined using ML-Relate, including only those relationships for which ‘unrelated’ was not also a likely option. (Note: variation in edge length is an artefact of the figure arrangement and does not convey information.) Social units with no missing adult members are indicated by an asterisk.

Out of the adult females, we identified eight pairs of individuals for which parent–offspring was the relationship with the highest likelihood. For six of these pairs, parent–offspring was the only relationship consistent with the genetic data at the 0.05 level of significance, but for two pairs, sibling relationships also met this level of significance. All of these parent–offspring pairs were within social units, rather than between them (figure 1). Average pairwise relatedness between mother–offspring pairs identified among adults was 0.50 (range: 0.43–0.59)

Pairs of adult females for which the most likely relationship was second degree (half-siblings/grandmother–granddaughter) were much more common (*n* = 43), but for the majority of these (74.4%), the genotypes were also consistent with the individuals being unrelated. For two inter-social unit pairs of individuals, full-siblings was the most likely relationship, but in both cases, the genotypes were also consistent with the individuals being second-degree relatives. Of these putative second-degree relationships, 88.9% were split across different social units. For the subset of 13 pairs for which ‘unrelated’ was not a probable relationship, all but one were between social units (figure 1). In two of these cases, the putative second-degree relatives had different mtDNA haplotypes, suggesting that any kinship between these individuals is paternal. For the remaining 10 well-supported sibling pairs between social units, it could not be readily distinguished whether they resulted from shared paternity or from maternal relatives (half-siblings or grandmother–granddaughter) splitting into separate social units. Average pairwise relatedness between putative second-degree relatives for which ‘unrelated’ was not a plausible option was 0.32 (range: 0.20–0.54).

### 3.5. Relatedness and haplotype sharing predicting association across all individuals

Across all known social unit members, association was significantly positively correlated with pairwise relatedness and with mtDNA haplotype sharing for all four measures of association examined (table 2). When the dataset was restricted to pairs of individuals in the same social unit, the correlations between pairwise relatedness and all scales of social association were positive and significant (table 2), though only marginally so for long-term close associations (i.e. clusters in a yearly sampling period).

**Table 2.** Correlation between measures of social association and pairwise relatedness (Rel) or mtDNA haplotype sharing (Hap) across all individuals (*n* = 55). Pairwise values for mother–calf pairs were excluded. This relationship was also tested after restricting to members of the same social unit. Association values were calculated using ‘both identified’ as the association index.

association definition	sampling interval	predictor	all individuals		the same social unit	
			matrix corr.	*p*-value	matrix corr.	*p*-value
day	year	Rel	0.200	<0.001	0.221	<0.001
		Hap	0.248	<0.001	—	
2 h	10 day	Rel	0.217	<0.001	0.187	0.003
		Hap	0.251	<0.001	—	
cluster	year	Rel	0.218	<0.001	0.130	0.035
		Hap	0.244	<0.001	—	
cluster	6 h	Rel	0.206	<0.001	0.197	0.001
		Hap	0.175	<0.001	—	

**Figure 2.**
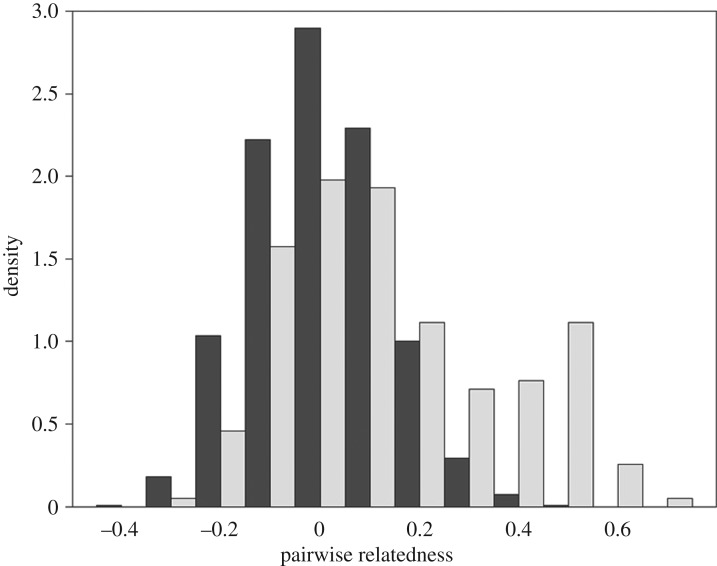
Distributions of pairwise relatedness values within (light grey) and between (dark grey) sperm whale social units. Relatedness values were calculated using Wang's estimator [62].

Members of the same social unit were also more closely related to each other than expected by chance (matrix correlation = 0.273, *p* < 0.001, *n* = 65). The mean relatedness between individuals in the same social unit was 0.139 (s.d.: 0.221, *n* = 200), whereas between individuals in different social units it was 0.004 (s.d.: 0.132, *n* = 1880). Relatedness values within social units were bimodally distributed, with a local maximum at approximately 0.5, and a global maximum just above zero (figure 2).

### 3.6. Relationships within social units

**Figure 3.**
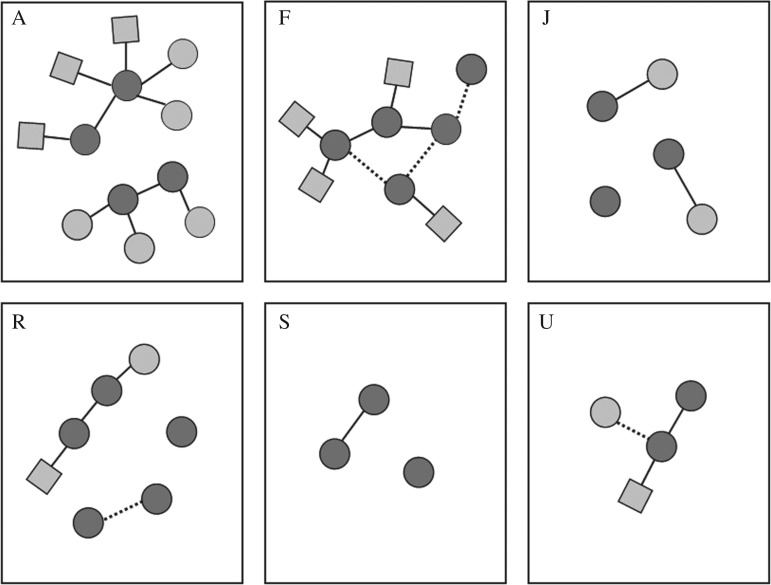
Relationship networks of well-sampled social units, based on genetic data. Females are indicated by circles and males by squares. Dark grey indicates adults and light grey indicates offspring. Solid lines denote mother–offspring relationships, as determined using Colony or ML-Relate. Dotted lines indicate pairs that were most likely second-degree relatives, but for which ‘unrelated’ was also a likely option (as determined using ML-Relate). Genetic data were unavailable for six offspring; these individuals are not shown. (Note: variation in edge length is an artefact of the figure arrangement and does not convey information.)

Within social units, parent–offspring relationships made up between 14.3 and 33.3% of relationships (16.9% overall), and between 0 and 33.3% of relationships were defined as second-degree relationships (15.5% overall), leaving between 33.3 and 80% of relationships as more distant than second degree, potentially including unrelated individuals (table 1). Most individuals (82.5%) had a mother or offspring in their social unit, and out of those who did not, the majority (57.1%) had a second-degree relative (figure 3). The remaining 7.5% of individuals had no relatives deemed to be first- or second-degree relatives sampled from their social unit.

For no social unit could all members be connected in a single network using only parent–offspring relationships, but for two social units, all members could be connected when second-degree relationships were included (figure 3). The remaining four social units had one or two missing connections between members, even when second-degree relationships were included (figure 3). In these social units, all unsampled members were calves, whose mothers were assumed (based on social data) to be among the sampled individuals, and so breaks in the genetic network are not likely due to the omission of these individuals, but could be due to deceased, unknown relatives.

### 3.7. Kinship predicting association within social units

For the two best-sampled large social units, association was statistically significantly and positively correlated with pairwise relatedness, at both sampling intervals (table 3). For the remaining four well-sampled social units, these correlations were non-significant and had mixed directions (table 3; electronic supplementary material, figure S1).

**Table 3.** Intra-unit social association preferences predicted by pairwise relatedness. Association was defined as identification in the same cluster, using ‘both identified’ as the association index. Pairwise values for mother-dependent calf pairs were excluded. Mantel tests were performed with 10 000 permutations.

social unit	*N*	sampling interval	matrix correlation	*p*-value
A	12	2 h	0.26	0.010
		day	0.45	<0.001
F	9	2 h	0.17	0.006
		day	0.11	0.012
J	5	2 h	−0.05	0.740
		day	0.12	0.529
R	7	2 h	−0.12	0.836
		day	−0.10	0.896
S	3	2 h	0.90	0.505
		day	0.99	0.164
U	4	2 h	−0.75	0.882
		day	−0.63	0.961

Two social modules were identified within unit A, the composition of which remained similar across years (though the strength of social modularity decreased across the years examined). In all years, the delineation of these social modules corresponded to the social unit's two matrilineal clusters of mother–offspring pairs (table 4).

**Table 4.** Social modules and strict matrilines in social unit A across time. Strict matrilines were defined based on mother–offspring relationships (figure 3). Social modules were based on association as clusters in a daily sampling period, using half-weight indices. Module composition is indicated by block shade, stippled shading indicates uncertainty in module assignment (|eigenvector| less than 0.1), and missing blocks indicate the individual was not seen (and presumably was not alive) in that year. Good divisions within a network are generally indicated by modularity values of roughly 0.3 or greater [68]. Per cent agreement with matrilines (% agreement) does not include uncertain module assignments.

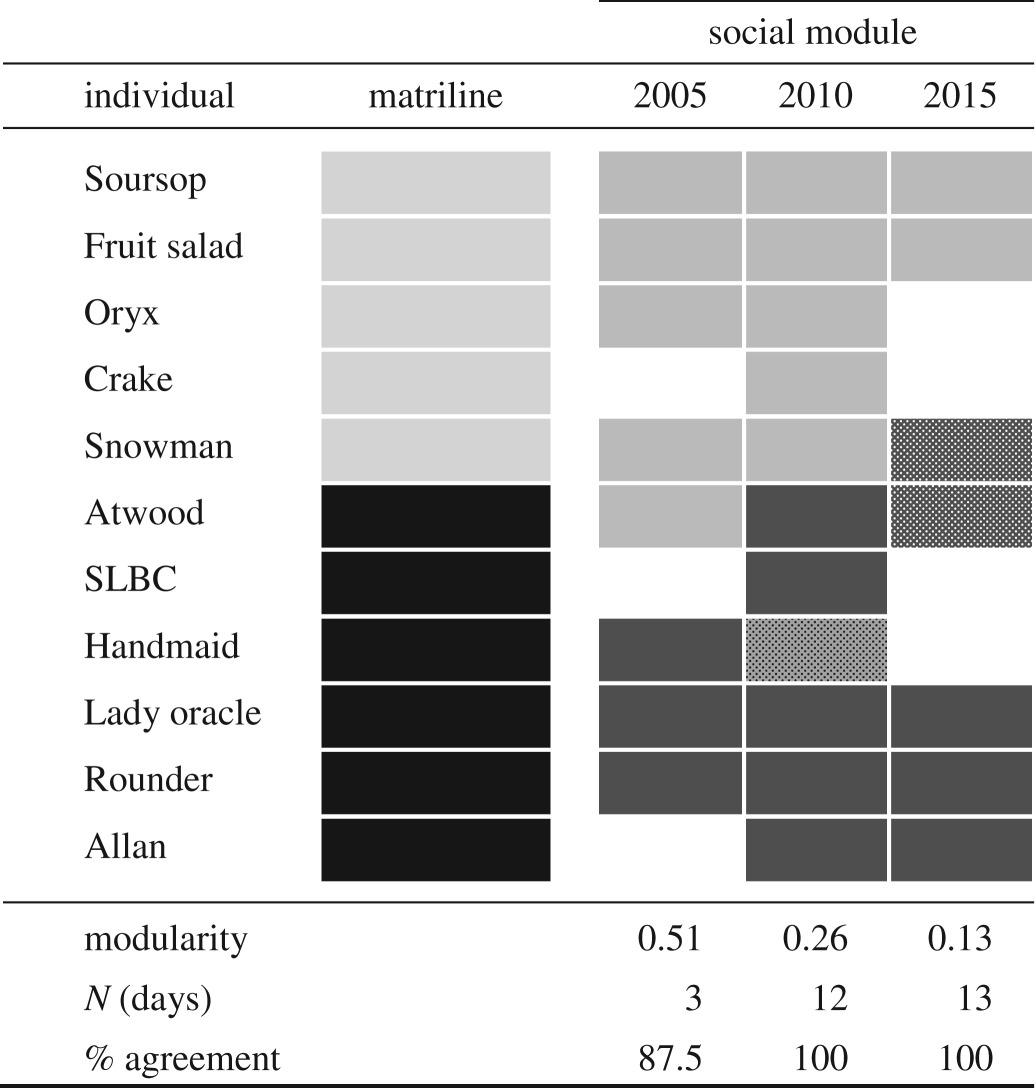

### 3.8. Kinship predicting association between social units

Association between social units was not significantly correlated with having a shared mtDNA haplotype at any level tested (*p* ≥ 0.17 for all four measures of association; table 5). Some pairs of social units with the same mtDNA haplotype never associated (e.g. units P and A, and units S and T), while units A and D, with differing haplotypes, frequently associated.

**Table 5.** Correlation between measures of inter-unit social association and mean pairwise relatedness (Rel) or mtDNA haplotype sharing (Hap). Association values were calculated using half-weight indices. The tests were repeated with the pairwise values for units U and F omitted (no UF). Mantel tests were performed with 10 000 permutations (*n* = 11).

association definition	sampling interval	predictor	all social units		no UF	
			correlation	*p*-value	correlation	*p*-value
day	year	Hap	0.33	0.17	0.32	0.11
		Rel	0.23	0.15	0.12	0.43
2 h	day	Hap	0.14	0.31	0.11	0.42
		Rel	0.13	0.33	−0.06	0.71
cluster	year	Hap	0.07	0.60	0.02	0.71
		Rel	0.25	0.09	0.09	0.48
cluster	2 h	Hap	0.13	0.24	0.09	0.38
		Rel	0.26	0.06	0.03	0.78

Association between social units, defined as being in a cluster together, seemed to be weakly correlated with mean relatedness for both sampling periods (table 5). For the coarser measures of association, association and mean relatedness between social units were not significantly correlated.

The marginally non-significant correlations between relatedness and fine-scale association were primarily driven by one pair of social units, U and F, which had the highest mean relatedness value of any pair of social units (mean relatedness = 0.112) and the highest association index at all levels of association. When the data point for this pair of social units is removed, the size and significance of all correlations dropped (table 5).

## 4. Discussion

This study improves upon what has been previously achieved by examining more social units, with a higher genetic resolution (38–80% more microsatellite loci), than previous studies [26–29], resulting in a uniquely detailed exploration of sperm whale kinship patterns in relation to social structure.

Kinship was clearly correlated with several measures of social association, particularly social unit membership (figure 2) and intra-social unit association preferences within two well-sampled social units (table 3), suggesting that kin-selection may indeed be a contributing driver of sperm whale social structure. However, substantial variation in patterns of associations remained unexplained by kinship, notably including association preferences between the majority of social units. Thus, other drivers of cooperation, such as group augmentation and reciprocal altruism, likely interact with kin-selection to drive the cooperative social structure we observe. Preferential cooperation between particular individuals or between particular social units could also be based in culture or personality, or some preferences may be by-products of circumstance and convenience.

We found a higher degree of relatedness and matrilineality in social units than has been reported in other regions [26,28,29]. Even so, it is unlikely that all of the social units that we examined were strict matrilines. The presence of a living common ancestor was not conclusively demonstrated in any social unit, despite all adult female members of these social units being genetically sampled. Rather, inference of one or two intermediary relatives that are dead or gone would be required in each social unit before the presence of a living common ancestor could be assumed (figure 3). Additionally, no social unit splits have been observed in 96 social unit-years of observation off Dominica [69]. This is despite a mean 4.5% per year decrease in the number of adults over the study period [69], which would predict the death of common living ancestors in roughly four social units across the study period. The presence of second-degree relationships between social units (figure 1) could indicate past social unit splits after the death of a common ancestor, but such relationships could also be explained by paternal relatedness. Indeed, paternal relatedness is the only explanation in cases where the second-degree relatives have different mitochondrial haplotypes (e.g. relationships between unit A and either unit J or F). The genetic data for all social units examined were consistent with our less stringent category, ‘generally matrilineal’. However, haplotype sharing does not necessitate close matrilineal co-ancestry, especially for the very common haplotype, A. As such, we could not rule out the possibility that the social units contained unrelated matrilines. Even so, social units composed of multiple matrilines would still be matrilineally based.

As our resolution of social data improves, so does our ability to investigate stability and distinguish constant companions from preferred associates. This is exemplified by unit A, which had all members genetically sampled, and was observed in seven different years between 2005 and 2016. This social unit was composed of two strict matrilines (figure 3), which were unrelated or separated by at least two absent intermediary relatives. This social unit was composed of two social modules that aligned well with the delineation of these two matrilines (table 4), indicating higher association within than between the strict matrilines in this unit. Based on our definition of social units (box 1), these individuals qualified as members of a single social unit, but the rate at which these two matrilines associated varied substantially across the study, and they were often observed apart (electronic supplementary material, table S3). Additionally, over the course of our study period, we documented the merger of two social units, U and F, which were originally classified as separate social units, using data as far back as 1995 [15]. From 2008 onwards, their association rate generally increased, such that by 2012 they were scarcely seen apart (electronic supplementary material, table S4). They met our definition (box 1) to be classified as a single social unit by 2009, even though at that point in their gradual merger the two social units were often seen apart (electronic supplementary material, table S4). This suggests that social unit members, as we have defined them, are not such constant companions as previously assumed, despite our definition of social units (based on [15]) being more stringent than or similar to what has been used in other studies [19,30,70,71]. Rather, it appears that, in some cases, sub-social unit social structures may exist but go undetected with the types of analyses currently used to define constant companions, which often rely on sparse data. It is not clear how sub-social unit social structures are actually expressed in the day-to-day life of a social unit at sea, perhaps by a separation of several kilometres between subunits, or perhaps by much greater distances.

A multi-level social structure, composed of nested layers of increasingly close kin, is not unique to sperm whales, but appears convergently in other marine and terrestrial mammals. Killer whale (*Orcinus orca*) social structure is also composed of several nested tiers, the smallest tier comprising matrilines of two to nine individuals that associate closely and constantly [72]. These matrilines then belong to higher-level groups. The association patterns between the within-social unit matrilines that we observed in social unit A are concordant with this social organization among killer whales. Sperm whales have very similar life histories and social systems to elephants [73]. Distributions of relatedness within and between sperm whale social units (figure 2) and within and between African elephant (*Loxodonta africana*) core social groups (see [74]—Fig. 1) are remarkably similar, suggesting similar degrees of matrilineality in their social structures. Also as in sperm whale social units, kinship predicts association within African elephant core groups [74] but kinship does not well predict higher levels of association, among either sperm whale social units or African elephant core groups [74].

Social and ecological contexts, such as the presence of dependent calves or limited resources, likely influence social structure and may encourage flexibility in how widely cooperation is extended beyond close kin. For example, changes in social context may have motivated the merger of units F and U because this merger occurred in parallel with changes in group size and composition (see electronic supplementary material, discussion and table S4). Social unit fusion has the potential to decrease the degree of matrilineality, depending on the relationship between the merging social units. In the case of units F and U, the merging social units were the most closely related pair of social units in our study, suggesting the possibility that mergers may preferentially occur between kin, which would minimize the breakdown of matrilineality. Similarly, among African elephants, core group splits and mergers are predicted by kinship [74].

The degree of relatedness within social units that we observed in the Eastern Caribbean is greater than those reported for sperm whale social units in the eastern tropical Pacific [26,29]. One potential reason for such differences in patterns of kinship and association is the degree to which populations were affected by modern whaling; sperm whales were much more heavily targeted in the eastern tropical Pacific than in the Caribbean [14]. Likewise, in African elephants, the relative importance of kinship to social structure was diminished in a more heavily poached population [75]. In African elephants, evidence suggests that individuals form associations with non-relatives if their relatives are poached [74,75], and the same is likely true for sperm whales [14].

Alternatively, these regional differences in the matrilineally based social structure of sperm whales may relate to differences in characteristics of prey affecting optimal group size [14], as appears to be the case among killer whales [76]. Two distinct ‘ecotypes’ of killer whales, known as ‘residents’ and ‘transients’, eat primarily fish and marine mammals, respectively, and have notably different matrilineal social structures [72]. Among ‘residents’ up to four generations are found in stable matrilineal groups, but among ‘transients’ smaller groups are found containing only one to two generations [72], seemingly because the smaller group size is more optimal for hunting the ‘transient' killer whales’ primary prey, the harbour seal (*Phoca vitulina*) [76].

Another factor affecting the social context of the individuals in this study is the population's current state of critical decline, with most social units losing members [69]. Social relationships can have fitness consequences, as in baboons, where female sociality correlates with reproductive success [77], and social structure influences processes like the transmission of disease or information [78]. The loss of individuals may also lead to changes to the matrilineal structure of sperm whale social units, perhaps encouraging mergers of unrelated social units, or discouraging splits of social units that have lost their common maternal ancestor, similar to the effect that whaling may have had on social structure in the Eastern Tropical Pacific [14]. As such, understanding the social structure of this population, and the genetic diversity underpinning it, is important from a conservation perspective. Understanding drivers of social structure can aid our understanding of how it may change when individuals are lost from this population.

## 5. Conclusion

While this study demonstrates that kinship is clearly an important factor influencing sperm whale social relationships, we also see that it is not the be-all and end-all. Social units were largely composed of kin but did not appear to be rigidly delineated by matrilines. Likewise, social associations within social units were biased towards closer relatives, but as a general trend, rather than a strict rule. Social and ecological context likely affect the degree of matrilineality in sperm whale social structure, leading to variation both within social units across time, and broadly across populations in different ocean basins. Overall, our findings support sperm whale society as being matrilineally based, but not strictly so; rather, it is nuanced and multi-faceted, resembling other complex matrilineal societies, such as among elephants and killer whales.

## Supplementary Material

Supplemental methods and association results

## Supplementary Material

Social association sample sizes

## Data accessibility

Data are available from the Dryad Digital Repository: (http://dx.doi.org/10.5061/dryad.63464hf) [79].
