# Supplementary material for: Kinship influences sperm whale social organization within, but generally not among, social units
Source: R Soc Open Sci. 2018 Aug 29;5(8):180914. doi: 10.1098/rsos.180914 (PMC6124104; doi:10.1098/rsos.180914)
Supplement: Supplemental methods and association results [file rsos180914supp.pdf]

Electronic Supplementary Material for: Konrad C, Gero S, Frasier T, Whitehead H (2018) Kinship influences sperm whale social organization within, but generally not among, social units. Royal Society Open Science.

Methods

Measuring Social Association

Across our study period, demographic changes affected our population, as individuals were born and died. Therefore, for most analyses, we used an association index, 'both identified', that minimizes the bias of these changes on association index values. Using only the sampling periods in which both individuals were identified, this index calculates the proportion of those sampling periods in which the individuals were associated [1]. However, this index typically requires long sampling periods to obtain enough periods within which both individuals were identified. For the analyses that would not be strongly affected by demographic changes, namely those within annual field seasons, and those at a social unit-level, rather than an individual-level, we used half-weight indices (HWI) of association [2]. This index best corrects for the types of biases in identification rates that are typical of cetacean photo identification [1,2].

To examine association preferences across different time scales, we used a variety of sampling periods, chosen depending on the definition of association and the association index used. The shortest period used was two hours, which corresponds to approximately two dive cycles in sperm whales and has been applied in other studies of this species [3,4]. With this sampling period, we aimed to maximize the number of samples while allowing ample opportunity for clusters to disband and clusters with new compositions to form. The longest period used was 'year', which has also been previously applied in this species [4] to highlight long-term associations, removing potential autocorrelation across sequential days.

Microsatellite Genotyping

All PCRs for microsatellite loci were carried out in 20 µl reactions in 1x PCR buffer, with 1.5 mM $MgCl_2$, 0.2mM of each dNTP, 0.3 µM of each primer, 0.05 U/µl of GoTaq Flexi DNA polymerase (Promega, Madison, WI) and 10 ng of template DNA (based on functional concentration, determined based on *ZFX/ZFY* gene fragment brightness). Reactions were run on an ABI Veriti 96 well thermal cycler (Applied Biosystems, Foster City, CA) with the following parameters: initial denaturing for 5 min at 94°C, then cycles of denaturation for 30 s at 94°C, annealing for 1 min, and extension for 1 min at 72°C, followed by a final elongation step, of either 10 min at 72°C or 45 min at 60°C. For locus-specific annealing temperatures and numbers of cycle, see Table S2. We included a no-template negative control with all reactions.

For four loci that did not amplify well with this standard procedure, a biphasic touchdown (TD) PCR protocol was used, to maximize amplification of low quality DNA while minimizing spurious amplification. This protocol consisted of a phase of TD-PCR [5], where annealing temperature (T_a) was started at 10°C above the final T_a and dropped by 0.5°C with each cycle, for 20 cycles, followed by 10 cycles at the final T_a. In a second phase of PCR, 2 µl of this first PCR product was used as template DNA, and the same cycle parameters were used as for the standard procedure.

To genotype the samples, we performed capillary electrophoresis to size separate and visualize the PCR product, using an ABI 3500xl Genetic Analyzer (Applied Biosystems, Foster City, CA). Before loading samples for genotyping, PCR products for some loci were diluted in distilled water (see Table S2 for dilution ratios), and up to three loci (that were labelled with different fluorescent molecules and had been amplified in

separate PCRs) were combined. We used the program GeneMarker (SoftGenetics, State College, PA) to automatically score fluorescence peaks, and all allele calls were confirmed manually by eye and then manually re-inspected a second time.

mtDNA haplotype sequencing

For the majority (80%) of sequencing reactions, initial PCRs were carried out in 20 µl reactions in 1x PCR buffer, with 1.5 mM $MgCl_2$, 0.2mM of each dNTP, 0.3 µM of each primer, 0.05 U/µl of GoTaq Flexi DNA polymerase and 10 ng of template DNA (based on functional concentration). Reactions were run on an ABI Veriti 96 well thermal cycler with the following parameters: initial denaturing for 5 min at 94°C, then cycles of denaturation for 30 s at 94°C, annealing for 1 min at 55°C, and extension for 1 min at 72°C, followed by a final elongation step, of 45 min at 60°C. Excess dNTPs and primers were digested in an enzymatic reaction containing 5 µl PCR product, 0.65µl Antarctic phosphatase buffer (50 mM Bis-Tris-Propane-HCl, 1 mM $MgCl_2$, 0.1 mM $ZnCl_2$, pH 6.0), 0.1µl Antarctic phosphatase (New England Biolabs, Ipswich, MA), and 0.03µl exonuclease I (New England Biolabs, Ipswich, MA). For this reaction, samples were incubated for 15 min at 37°C, followed by 15 min at 80°C. Sequencing reactions, using the product from the preceding reaction, were then carried out in 15µl reactions using the BigDye Terminator v3.1 Cycle Sequencing kit (Applied Biosystems, Foster City, CA), containing 1.5µl of Reaction Mix, 3µl of Sequencing Buffer, and 1µl (at 10 µM) of the primer t-Pro [6]. Reactions were run on an ABI Veriti 96 well thermal cycler with the following parameters: initial denaturing for 2 min at 96°C, then cycles of denaturation for 20 s at 96°C, annealing for 20 sec at 50°C, and extension for 4 min at 60°C.

The remaining 20% of reactions were carried out using a BigDye® Direct Cycle Sequencing Kit and the accompanying protocol (Applied Biosystems, Foster City, CA), which used M13 tailed primers.

After the sequencing reaction, salts, nucleotides and primers were removed via ethanol precipitation [7] and resuspended in 10µl of HiDi formamide (Applied Biosystems, Foster City, CA). We included a no-template negative control with all reactions, and 14 samples were duplicated as blind replicates to estimate the consistency of haplotype sequencing. The PCR products were visualized using an ABI 3500xl Genetic Analyzer. Sequences were manually trimmed and edited using 4Peaks (nucleoytes.com) and were manually aligned using BioEdit 7.2.5 [8].

Table S1. Reasons for rejection of microsatellite loci that were excluded from analysis.

Locus	Rejection Reason	Reference
EV14Pm	Amplified poorly	Valsecchi & Amos (1996) Mol Ecol 5:151-156
FCB10	Amplified poorly	Buchanan et al. (1996) Mol Ecol 5:571-575
FCB4	Amplified poorly	Buchanan et al. (1996) Mol Ecol 5:571-575
FCB5	Amplified poorly	Buchanan et al. (1996) Mol Ecol 5:571-575
FCB6	Amplified poorly	Buchanan et al. (1996) Mol Ecol 5:571-575
GATA028	Amplified poorly	Palsboll et al. (1997) Mol Ecol 6:893-895
GATA098	Failed to amplify	Palsboll et al. (1997) Mol Ecol 6:893-895
GT023	Amplified poorly	Berube et al. (2000) Mol Ecol 9:2181-2183
IGF1	Unrelaible genotyping	Barendse et al. (1994) Nat Genet 6:227-235
RW31	Amplified poorly	Waldick et al. (1999) Mol Ecol 8:1763-1765
RW48	Amplified poorly	Waldick et al. (1999) Mol Ecol 8:1763-1765
TEXVET19	Unrelaible genotyping	Rooney et al. (1999) J Heredity 90:228-231
TR3A1	Amplified poorly	Frasier et al. (2006) Mol Ecol Notes 6:1025-1029
TR3F2	Failed to amplify correct fragment	Frasier et al. (2006) Mol Ecol Notes 6:1025-1029
TR3F4	Amplified poorly	Frasier et al. (2006) Mol Ecol Notes 6:1025-1029

Table S2. Locus-specific microsatellite PCR protocols and results. For the biphasic protocol, initial annealing temperatures for the first phase started 10˚C above T_a and dropped by 0.5°C with each cycle, for 20 cycles, followed by 10 cycles at the final T_a, and the second phase used same T_a and 30 cycles. Final elongation temperature was either (A) 60˚C for 45 minutes or (B) 72˚C for 10 minutes. PCR product was diluted, in distilled water, according to dilution ratio, prior to capillary electrophoresis.

							Protocol					
Locus	Na	Ho	N	Allele range (bp)	Repeat type	Reference	T_a (˚C)	Cycles	Biphasic	Final Elongation	Dilution Ratio	Dye Label
D08	6	0.716	95	80-102	Di	Shinohara et al. (1997) Mol Ecol 6: 695-696	55	30		A		NED
D22	6	0.684	95	107-117	Di	Shinohara et al. (1997) Mol Ecol 6: 695-696	55	30		A		PET
EV104Mn	5	0.653	95	152-160	Di	Valsecchi & Amos (1996) Mol Ecol 5:151-156	55	30		B		6-FAM
EV1Pm	11	0.621	95	109-141	Di	Valsecchi & Amos (1996) Mol Ecol 5:151-156	58	30		B		NED
EV5Pm	9	0.691	94	146-168	Di	Valsecchi & Amos (1996) Mol Ecol 5:151-156	60	32		B		VIC
EV94Mn	12	0.779	95	195-223	Di	Valsecchi & Amos (1996) Mol Ecol 5:151-156	55	32		B		6-FAM
FCB1	10	0.916	95	117-137	Di	Buchanan et al. (1996) Mol Ecol 5:571-575	55	30	Y	B	1:9	VIC
FCB14	12	0.779	95	279-311	Di	Buchanan et al. (1996) Mol Ecol 5:571-575	55	39		B		VIC
FCB17	17	0.926	95	135-183	Di	Buchanan et al. (1996) Mol Ecol 5:571-575	55	35		B		6-FAM
FCB3	11	0.789	95	134-154	Di	Buchanan et al. (1996) Mol Ecol 5:571-575	55	37		B		VIC
GATA417	3	0.516	93	169-185	Tetra	Palsboll et al. (1997) Mol Ecol 6:893-895	55	30		B		PET
MK6	7	0.625	88	144-166	Di	Krützen et al. (2001) Mol Ecol Notes 1:170-172	60	37		B		VIC
RW34	13	0.916	95	84-110	Di	Waldick et al. (1999) Mol Ecol 8:1763-1765	50	32		A		6-FAM
SW10	11	0.853	95	136-158	Di	Richard et al. (1996) Mol Ecol 5:313-315	60	30	Y	B	1:9	NED
SW13	10	0.895	95	131-171	Di	Richard et al. (1996) Mol Ecol 5:313-315	50	30		A		6-FAM
SW2	6	0.553	94	62-76	Di	Richard et al. (1996) Mol Ecol 5:313-315	55	37		A	1:24	NED
TEXVET5	11	0.809	94	192-214	Di	Rooney et al. (1999) J Heredity 90:228-231	55	30	Y	B; A	1:1	NED
TR3G2	7	0.789	95	159-183	Tetra	Frasier et al. (2006) Mol Ecol Notes 6:1025-1029	55	30		A		PET
Mean	9.3	0.75	94.3									

Supplemental Discussion of Social Context and Social Structure

In social unit A, variation in association rates between the two matrilineal families did not have a clear relationship with changes in unit composition, but the year with the highest rate of association did correspond with the presence of two new calves (Table S3).

Notable increases in association rates between social units U and F correspond with the first observations of a new calf in social unit F in 2008, the loss of two adult members from social unit F in 2011, and the departure of a juvenile male from each social unit in 2012 (Table S4).

Table S3. Changing rates of association within Unit A, which was composed of two strict matrilines (A1 and A2), and changing unit composition across time. Half-weight index (HWI) values used association as observation of the A1 matriline and the A2 matriline within 2h, within a daily sampling period. Members were classified as adults (A), which included juvenile males, or as calves (C). Neither matriline was observed in 2006, 2007, 2011 or 2012.

Year	Days Obs		HWI	Unit Composition	
	A1	A2		A1	A2
2005	3	2	0.80	4A	2A 2C
2008	5	9	0.43	4A 1C	3A 1C
2009	4	4	0.50	4A 1C	3A 1C
2010	11	10	0.86	4A 2C	3A 2C
2014	0	2	--	--	3A
2015	12	12	0.75	4A	3A
2016	16	1	0.12	4A 1C	--
Total	51	40	0.57		

Table S4. Changing rate of association between Units U and F, and changing unit composition across time. Half-weight index (HWI) values used association as observation of Unit U and Unit F within 2h, within a daily sampling period. Members were classified as adults (A), which included juvenile males, or as calves (C). Neither social unit was observed in 2014.

Year	Days Obs		HWI	Unit Composition	
	F	U		F	U
2005	40	0	--	6A 1C	--
2006	9	1	0.00	5A 1C	3A 1C
2007	7	0	--	5A 1C	--
2008	20	12	0.69	5A 2C	3A 1C
2009	7	3	0.60	5A 2C	3A 1C
2010	14	11	0.80	5A 2C	3A 1C
2011	4	5	0.89	4A 1C	4A
2012	2	2	1.00	3A 1C	3A
2015	11	12	0.96	3A 1C	3A
2016	3	3	1.00	2A 1C	3A
Total	117	49	0.53		

Supplementary Figure

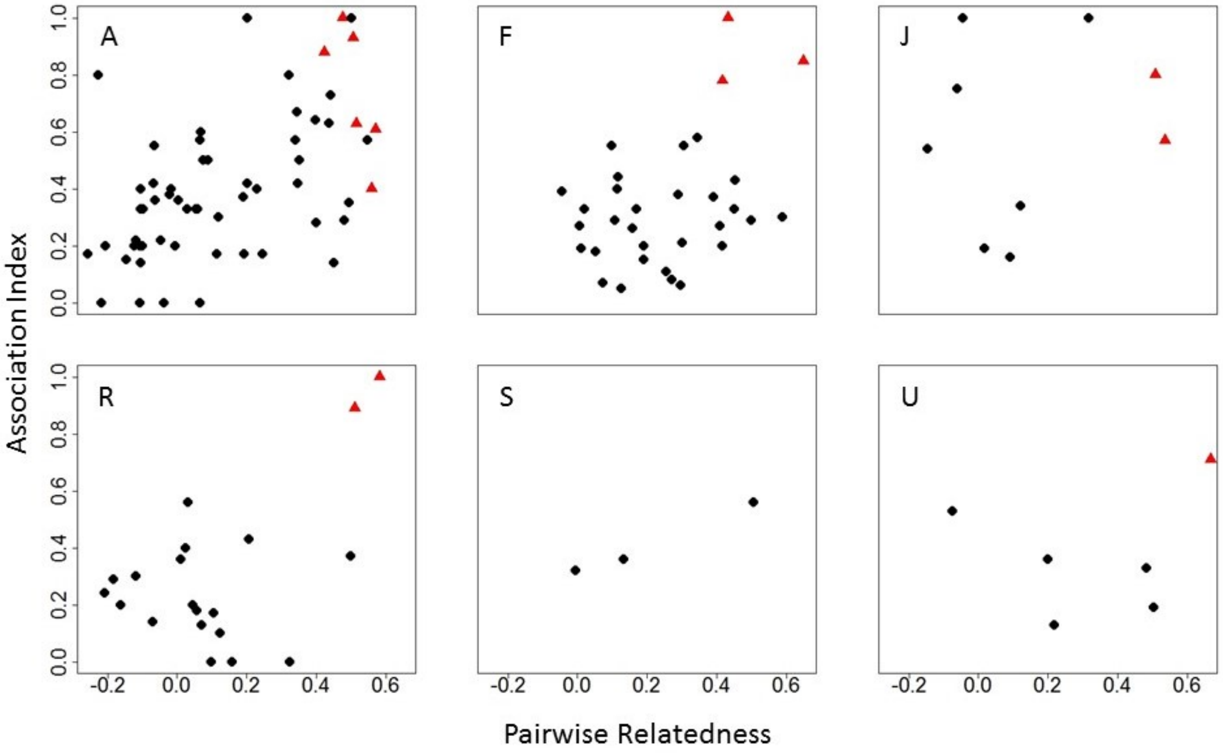

Figure S1. Intra-unit social association preferences predicted by pairwise relatedness. Association was defined as identification in the same cluster in a day, using 'both identified' to calculate the association index. Relatedness values were calculated using Wang's (2002) estimator [9]. Mother-dependant calf pairs are indicated by red triangles and were not included in the statistical analyses. Letters denote social unit.

Works cited

1. Whitehead H. 2008 *Analyzing Animal Societies*. Chicago, IL: The University of Chicago Press.

2. Cairns SJ, Schwager SJ. 1987 A comparison of association indices. *Anim. Behav.* **35**, 1454–1469.

3. Christal J, Whitehead H. 2001 Social affiliation within sperm whale (*Physeter macrocephalus*) groups. *Ethology* **107**, 323–340.

4. Gero S, Gordon J, Whitehead H. 2015 Individualized social preferences and long-term social fidelity between social units of sperm whales. *Anim. Behav.* **102**, 15–23. (doi:10.1016/j.anbehav.2015.01.008)

5. Korbie DJ, Mattick JS. 2008 Touchdown PCR for increased specificity and sensitivity in PCR amplification. *Nat. Protoc.* **3**, 13–15. (doi:10.1038/nprot.2008.133)

6. Yoshida H, Yoshioka M, Shirakihara M, Chow S. 2001 Population structure of finless porpoises (*Neophocaena phocaenoides*) in coastal waters of Japan based on mitochondrial DNA sequences. *J. Mammal.* **82**, 123–130.

7. Irwin DL, Mitchelson KR, Findlay I. 2003 PCR product cleanup methods for capillary electrophoresis. *Biotechniques* **34**, 932–936.

8. Hall TA. 1999 BioEdit: a user-friendly biological sequence alignment editor and analysis program for Windows 95/98/NT. *Nucleic Acids Symp. Ser.* **41**, 95–98. (doi:citeulike-article-id:691774)

9. Wang J. 2002 An estimator for pairwise relatedness using molecular markers. *Genetics* **160**, 1203–1215.
